# Analysis of hospital admissions due to accidental non-fire-related carbon monoxide poisoning in England, between 2001 and 2010

**DOI:** 10.1093/pubmed/fdv026

**Published:** 2015-03-09

**Authors:** Rebecca E. Ghosh, Rebecca Close, Lucy J. McCann, Helen Crabbe, Kevin Garwood, Anna L. Hansell, Giovanni Leonardi

**Affiliations:** 1 UK Small Area Health Statistics Unit, MRC-PHE Centre for Environment and Health, Department of Epidemiology and Biostatistics, School of Public Health, Imperial College London, London W2 1PG, UK; 2 Department of Epidemiology, Centre for Radiation, Chemical and Environmental Hazards, Public Health England, Didcot, Oxon OX11 0RQ, UK; 3 Centre for Radiation, Chemical and Environmental Hazards, Public Health England, Didcot, Oxon OX11 0RQ, UK; 4 London School of Hygiene and Tropical Medicine, London WC1E 7HT, UK

**Keywords:** carbon monoxide poisoning, epidemiology, hospital admissions

## Abstract

**Background:**

Accidental non-fire-related (ANFR) carbon monoxide (CO) poisoning is a cause of fatalities and hospital admissions. This is the first study that describes the characteristics of ANFR CO hospital admissions in England.

**Methods:**

Hospital Episode Statistics (HES) inpatient data for England between 2001 and 2010 were used. ANFR CO poisoning admissions were defined as any mention of ICD-10 code T58: toxic effect of CO and X47: accidental poisoning by gases or vapours, excluding ICD-10 codes potentially related to fires (X00-X09, T20-T32 and Y26).

**Results:**

There were 2463 ANFR CO admissions over the 10-year period (annual rate: 0.49/100 000); these comprised just under half (48.7%) of all non-fire-related (accidental and non-accidental) CO admissions. There was seasonal variability, with more admissions in colder winter months. Higher admission rates were observed in the north of England. Just over half (53%) of ANFR admissions were male, and the highest rates of ANFR admissions were in those aged >80 years.

**Conclusion:**

The burden of ANFR CO poisoning is preventable. The results of this study suggest an appreciable burden of CO and highlight differences that may aid targeting of public health interventions.

## Introduction

Carbon monoxide (CO) is a common, potentially fatal, colourless, odourless and tasteless gas that results from the incomplete combustion of fuels. CO exposure is a cause of fatalities and hospital admissions from accidental poisoning.^1–3^ Symptoms can range from subtle headaches to persistent neurological effects and death, depending on both the level and duration of exposure. Exposure can be both intentional and accidental. Accidental exposure can be from a variety of sources, including fires and domestic appliances such as boilers.^4^ Accidental poisoning not caused by exposure to fires is almost entirely preventable through the correct installation and maintenance of CO-emitting devices and the use of CO detectors.

Current Department of Health estimates for accidental CO poisoning in England suggest that there are 40 deaths, 200 hospital admissions and 4000 Accident and Emergency Department consultations annually.^5^ While these figures cover all causes of accidental CO admission, they may be underestimates of the true burden.^6–8^ This is due to a number of reasons including: misdiagnosis due to the non-specific symptoms such as headache, tiredness and nausea; non-confirmation of diagnosis on hospital records and death certificates, which may be due to a lack of awareness of CO poisoning by clinicians; and difficulty confirming exposure if the patient has been given oxygen or there are delays in blood testing (CO has a short half-life, usually between 4 and 6 h).^9,10^

Mortality due to accidental non-fire-related (ANFR) CO poisoning in the UK has been previously studied^3,11^ with Fisher *et al.* finding 40 annual accidental CO deaths between 2001 and 2010. There have been few studies of non-fatal accidental CO poisoning in the UK^1,4,10^ and none looking at hospital admissions on a national level. Papers published in France and the USA have highlighted the need for and feasibility of CO surveillance.^12–15^ Although data sources exist in the UK which could contribute to surveillance of CO,^16–18^ there is currently no established surveillance system.

This aim of this paper is to quantify the morbidity burden due to ANFR CO poisoning in England in (i) the context of other admissions to hospital for CO poisoning and (ii) to describe these admissions by region, sex, age and deprivation. This can be used as a baseline for future routine surveillance of CO morbidity and help target education and other interventions to reduce CO exposures.

## Methods

This study used Hospital Episode Statistics (HES) inpatient data for England between 2001 and 2010, held by the UK Small Area Health Statistics Unit (SAHSU). HES data form part of an administrative data set that records all admissions for NHS hospitals and facilities funded by the NHS in England.^19^ The data have undergone extensive cleaning of invalid values with further checks carried out by SAHSU to look at the completeness of fields and consistency of coding. Private hospitals account for a small proportion of hospital care in the UK, mostly dealing with elective admissions and it is likely that almost all admissions for CO poisoning will be captured within HES. On admission to hospital, patients are assigned to a consultant who is responsible for their treatment, and within the HES data set, a period of care under a consultant is termed an ‘episode’. This analysis was restricted to diagnoses on first episode of care (‘episode order’ equal to one) of a non-elective admission to hospital. For the period studied, 2001–10 inclusive, diagnoses were recorded using the International Classification of Diseases tenth revision (ICD-10). Each episode contains a primary diagnosis field as well as up to 19 secondary diagnosis fields (14 before April 2007 and 7 before April 2002) in which the reasons for admission are recorded.^20^

An admission for CO poisoning was counted as any diagnosis (primary or secondary) coded to ICD-10 code T58: toxic effect of CO. Accidental CO poisoning was defined as:

- T58 plus any mention of external cause code X47 (accidental poisoning by and exposure to other gases and vapours).

To provide context, accidental CO poisoning was compared with the proportions of (i) intentional CO poisoning and (ii) those where the intent was unknown:

1. T58 + X67: intentional self-poisoning by and exposure to other gases and vapours
2. T58 + Y17: poisoning by and exposure to other gases and vapours, undetermined intent or no additional external cause code.

ANFR CO poisoning was defined as accidental CO poisoning (T58 + X47) after excluding the following codes:

– X00-X09: exposure to smoke, fire and flames
– T20-T32: burns and corrosions
– Y26: exposure to smoke fire and flames, undetermined intent

The total numbers and proportion of accidental CO admissions were compared with other CO admissions for each sex and differences were tested using the *χ*^2^ test. This was done with and without the fire-related codes to determine what effect excluding these codes has on the totals.

Crude rates for each sex by intent of CO poisoning were calculated over time using mid-year population estimates for England from the Office for National Statistics (ONS). Rates of ANFR CO poisonings were analysed by calendar year. ANFR CO poisoning between 2001 and 2010 was investigated by month, rates by age group (using mid-year population estimates split by age) and deprivation. Deprivation was considered using the Carstairs index^21^ as this was the most appropriate for the time period considered. Admissions were also investigated by an alternative deprivation measure the Index of Multiple Deprivation 2007 (IMD) but as this presented a similar pattern we do not present the data. Using SAHSU's Rapid Inquiry Facility (RIF),^22^ indirectly age-standardized rates of ANFR CO poisonings were calculated by region using the population of England as a reference. The empirical Bayes smoothed relative risk of ANFR CO poisoning was calculated at local authority/district level using England as the reference.^22^ The relative risks were mapped both with and without adjustment for deprivation.

## Results

In the 10 years between 2001 and 2010, we identified 5312 total admissions to hospital for CO poisoning in England, of these 47% (2500 admissions) were for accidental poisoning (Table 1). Of the total 5312, it was not possible to determine the intent of 638 CO admissions (12%) as no intent code was provided (X47 or X67). Of these 638, 89% were not even provided with the ‘Unknown intent’ code Y17. After the exclusion of the fire-related external cause codes (250 records), there were 5062 NFR CO admissions for the period of study. The majority of the fire-related records excluded (210 records, 84%) were from the ‘Unknown’ category. There were 2463 ANFR CO admissions (48.7% of all NFR CO admissions) over the 10 years giving an annual rate of 0.49 per 100 000, 53% were of these were male.

**Table 1:** Carbon monoxide admissions (2001–10, England) by sex and intent

	*Accidental-X47, *n* (%)*	*Intentional-X67, *n* (%)*	*Unknown,^a^*n* (%)*	*Total *n* (%)*
	Including fire-related codes			
Female	1184 (47.4)	389 (17.9)	265 (41.5)	1838 (34.6)
Male	1316 (52.6)^b^	1785 (82.1)^b^	373 (58.5)^b^	3474 (65.4)
Total	2500 (100)	2174 (100)	638 (100)^c^	5312 (100)
	Excluding fire-related codes			
Female	1164 (47.3)	389 (17.9)	174 (40.7)	1727 (34.1)
Male	1299 (52.7)^b^	1782 (82.1)^b^	254 (59.3)^d^	3335 (65.9)
Total	2463 (100)	2171 (100)	428 (100)^e^	5062 (100)

^a^Combination of “T58 + Y17—Undetermined intent” and T58 with no intent code provided.

^b^Difference between sexes statistically significant : *χ*^2^ *P* < 0.001.

^c^Of these, 571were admissions with T58 but no other intent code.

^d^Difference between sexes statistically significant : ^d^*P* = 0.003.

^e^Of these, 362 were admissions with T58 but no other intent code.

Using only the primary diagnosis on admission, instead of any mention in the primary or secondary diagnosis fields, would have identified 15% (800 admissions) fewer CO admissions overall, but with identical proportions of male and female admissions. However, there were slightly more ANFR admissions (53% using primary diagnosis only versus 49%) (Supplementary data, Appendix).

**Fig. 1:**
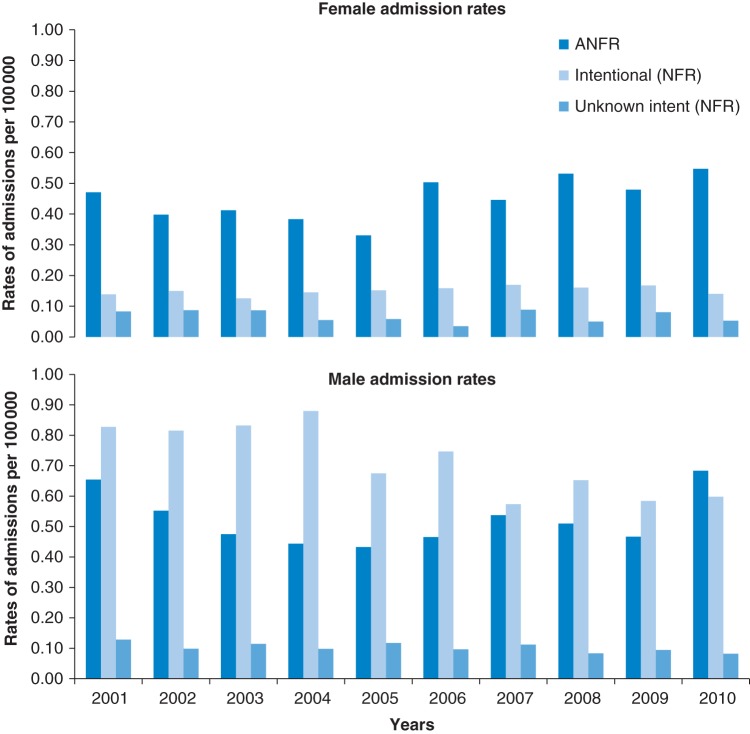
Non-fire-related (NFR) CO hospital admission rates over time. Denominator—ONS mid-year population estimates for England.

Figure 1 shows ANFR CO admission rates by year for England, split by cause and sex, compared with intentional and unknown intent admissions. Patterns by year for ANFR CO poisoning were similar in both female and male admissions; the rate of admissions decreased then increased across the years. The rate of intentional admissions showed a decrease in men but remained constant in women over the 10 years. The rate of admissions with unknown intent showed a slight decrease over the 10 years in men or women over time.

**Fig. 2:**
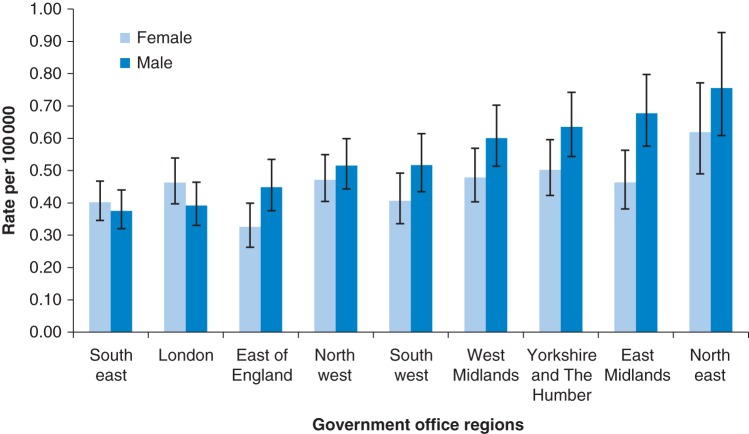
ANFR CO hospital admission rates per 100 000 person years for by government office region 2001–10. Missing GOR information for 2% of admissions (2.5% of male, 1.6% of female).

Figure 2 shows the admission rates for ANFR CO poisoning by government office regions in England with 95% confidence interval bars. In both males and females, there was a north–south difference with generally higher rates of accidental CO admissions (excluding fire-related codes) in northern regions of England compared with southern regions. The female rate was consistently lower than the male rate in most regions except London and the South-East. The region of England with the highest age-standardized rates in both males and females was the North-East (females 0.62, males 0.76 per 100 000 person years). The region with the lowest rate for females was the East of England (0.33/100 000) and for males it was the South-East (0.38/100 000).

The north–south pattern of risk within England was less clear by district, a finer spatial resolution (Supplementary data, Appendix). This persisted despite the relative risks being smoothed to allow for small numbers and was insensitive to adjustment for deprivation.

**Fig. 3:**
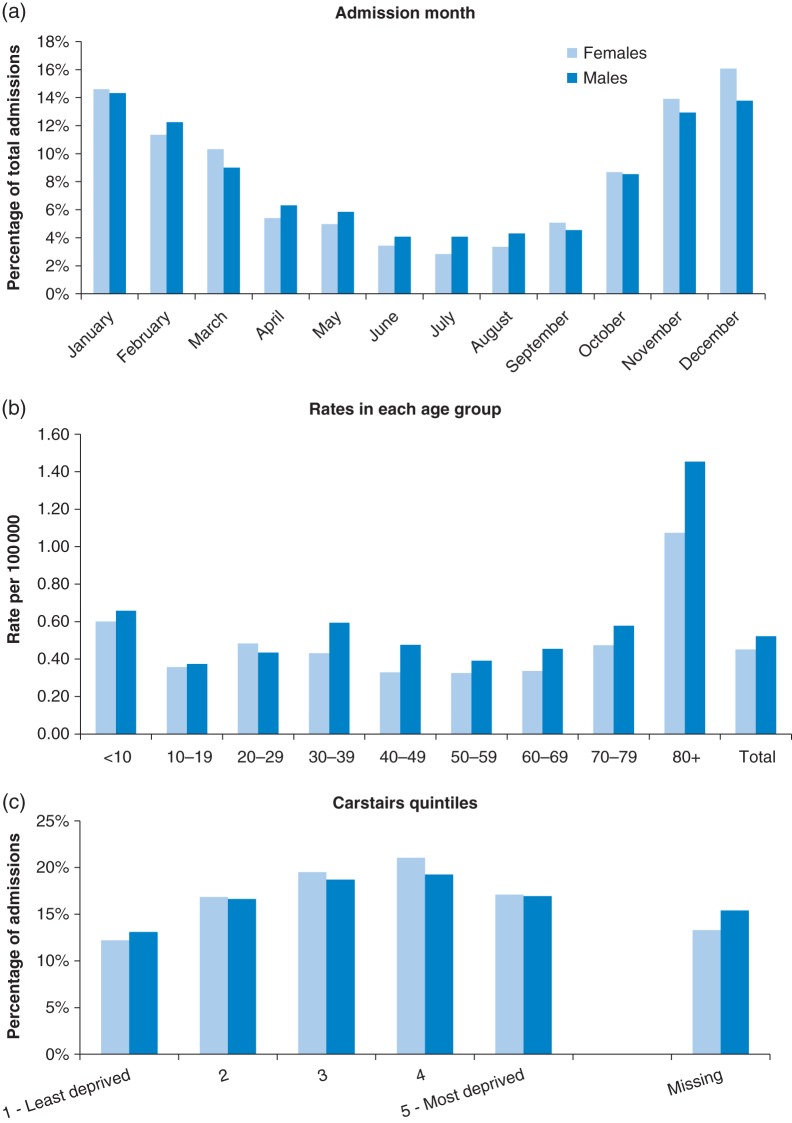
ANFR CO hospital admissions by (a) month of admission, (b) age groups and (c) Carstairs quintiles.

There was a clear pattern of increased admissions for ANFR CO poisoning in the colder months of November to February and decreased admissions in the warmest months of June to August (Fig. 3a). The highest percentage of admissions occurred in December (females 16.1%, males 14.3%) and the lowest percentage in June/July (July females 2.8%, June and July males 4.1%).

The data split by age group shows that the rates per 100 000 of ANFR CO poisonings were highest in the oldest age group (80+ years), this was particularly the case for male admissions (Fig. 3b). The youngest age group (<10 years) had the next highest rates of admissions over the 10 years for both sexes.

For both males and females, the proportion of ANFR CO admissions was lowest in those living in the least deprived areas, according to Carstairs index of deprivation^23^ (12.2% females, 13.1% males) (Fig. 3c), and increased with increasing area-level deprivation with a small decrease in the most deprived quintile. However, information on deprivation of area was missing for 15.4% of male and 13.3% of female admissions due to missing detailed geographical identifiers (*n* = 355). This does not affect the regional level comparisons as most (98%) admission records had a broad geographical identifier (region) even when missing more detailed geographical information.

## Discussion

### Main finding of this study

This is the first national study to attempt to quantify and describe the burden of ANFR CO hospital admissions in England. Between 2001 and 2010, there were 2463 ANFR CO admissions in England which was nearly half of all CO admissions to hospital. These admissions were approximately evenly split between males and females. This study found higher rates of ANFR admissions in the north of England, with more admissions in colder winter months. This study found a pattern of increasing admissions with increasing deprivation with the exception of the most deprived quintile.

### What is already known on this topic

Our annual average of 250 ANFR CO admissions per year in England was larger than the current estimates from the Department of Health of ∼200 per year.^5^ Our rate of admissions for ANFR CO poisoning was smaller than a study in the West Midlands (0.49 versus 1.1 per 100 000).^1^ However, Wilson *et al.* used an earlier time period (1988–94) and did not exclude fire-related admissions. We found a slightly higher rate of ANFR admissions than a study from Florida using hospital discharge records (0.32 per 100 000) over a similar time period to our study (1999–2007).^24^

The annual number of deaths from accidental CO poisoning in England and Wales over the same time period as our study (2001–10) has been estimated using coroner's reports obtained from ONS by Fisher *et al.* at 40 per year.^3^ This is similar to the 39 annual deaths estimated for the UK for an earlier period (1996–2007) using data from the CO-Gas Safety Society (COGSS) database.^11^ As expected the burden of non-fatal accidental CO poisoning in England is higher than burden from mortality. We found 250 annual accidental CO hospital admissions in England suggesting that ∼16% of admissions may result in mortality. This is greater than the proportion found by Fisher *et al.* who found 12.7% of CO deaths were due to ANFR poisoning between 2001 and 2010.^3^

### What this study adds

The greatest proportion of all English CO poisoning admissions were accidental (47%), similar to the proportion of accidental admissions seen in the West Midlands (43%)^1^ but larger than the proportion observed in Florida (33%).^24^ Accidental CO poisoning is likely to increase as a proportion of the total CO morbidity burden as admissions for intentional CO poisoning have been decreasing over the decade. This reflects the changes in suicide methods in England over the last decade with a reduction in suicides from CO poisoning by car exhaust gas as more cars were fitted with catalytic converters reducing CO exhaust emissions.^25^ ANFR admissions were statistically slightly more likely to be male (53 versus 47%), but this difference was much less marked than for ANFR mortality^3,11^ where 82% of deaths were male in the ONS data.^3^

Regional variation suggested that the North-East had the highest rates of admissions for ANFR CO poisoning, and there appeared to be a north/south divide in England with generally lower rates in the south. This observation may be due to generally colder temperatures in the North of England and possibly a greater use of domestic heating with solid fuels or gas. In most regions, women had lower rates of admissions than men except for London and the South-East where women had higher rates of admissions. This may be related to differences in the ethnic composition of London and surrounds, which are generally very different to the rest of the country,^26^ and consequently differences in heating and cooking practices. It was not possible to investigate this further as ethnic information in the HES data was limited with over 30% of the records missing this information.

There was a seasonal pattern in the ANFR CO admissions, with fewer admissions in the summer months versus the winter months, likely related to use of domestic heating with solid fuels or gas. This is consistent with a number of studies that have investigated the characteristics of CO poisoning in Iran, the USA and Europe.^1,24,27–33^ In general, these studies also found that CO poisonings are higher in the winter months (November–January), and this is also consistent with seasonal patterns in mortality from accidental CO admissions in the UK.^11^ This trend may provide an explanation for the observed pattern of ANFR CO admission rates that appeared to be decreasing in the first half of the decade but from 2006 onwards appeared to increase again. The three coldest time average winters^34^ in this decade were in 2006, 2009 and 2010 which was consistent with a rise in the ANFR CO admission rate.

For both men and women, the oldest (80+ years) age group had the highest rates of ANFR CO admissions followed by the youngest age group (<10 years) which is consistent with other studies.^1,3,24^ Younger than school age children and older retired adults may be expected to spend more time indoors and therefore have the most potential exposure to defective household fuel appliances such as boilers and cookers. They may also the most vulnerable to the effects of CO poisoning^1^ and may be acting as markers of more potential CO poisoning in the home as other individuals may be affected but not exhibit severe enough symptoms to be admitted to hospital. A study of admissions to English emergency departments also found a slightly higher proportion of cases in children; however, they did not see this in older people.^10^

There was a pattern of increasing admissions with increasing deprivation with the exception of the most deprived quintile. The trend seen may reflect a greater use of social housing by the most deprived group as well as legislation and health promotion, which tends to focus on the most deprived.^1^ Organizations responsible for providing social housing have legal requirements to perform gas safety checks which may have prevented ANFR CO poisoning in social housing tenants. Other explanations for the observed pattern may relate to missing information on area of residence (∼15% of all records) differentially occurring in most deprived groups. The Carstairs index is also an area-level measure of deprivation and as such may misrepresent the deprivation level of the individuals admitted to hospital.

### Limitations of this study

This study uses routine HES data and is subject to the usual limitations of using record-based systems, including differences in accuracy of diagnosis and recording and coding of data. The majority of records did have an external cause code, and after excluding fire-related codes, only 9% of records could not have a cause of CO poisoning determined and may have led to an underestimation of the number of ANFR CO admissions. Due to the non-specific symptoms of CO poisoning, the number of hospital admissions in HES is likely to be underestimated. In addition, many individuals with symptoms of non-acute CO poisoning may present first at primary care services rather than as a hospital admission leading to an underestimation of the risk of CO poisoning when using HES only. A study in four emergency departments in England suggested that only 20% of attendances later thought possibly to have been caused by exposure to CO were initially suspected and investigated as such.^10^ A similar pattern of CO underdiagnosis may apply to those admitted with common symptoms such as chest pain, difficulty breathing and headache.

We attempted to capture as many admissions as possible by considering both primary and secondary diagnoses. This decision increased the number of admissions we identified (compared with using primary diagnosis) by around 15%. Underdiagnoses of CO poisoning may also explain why we found such a small proportion (4.7%) of CO admissions that were potentially fire related, this is much lower than the proportion of CO deaths that has been related to fires (18.8%) in a study of 11 European Member States.^35^ Many fatal cases of fire-related CO poisoning, which may be picked up in a detailed coroner's report, could be missed at admission to hospital and therefore not be recorded in HES. In addition, CO poisoning may have been missed on admission to hospital especially if there are many other fire-related injuries.

Information on patient characteristics is limited and often incomplete or lacking in detail. HES records do contain ethnicity data, but the quality of the information is sometimes questionable with over a quarter of records that were examined not having this information. HES data were not linked with mortality data so we do not know how many individuals died from CO poisoning. However, further work to investigate this could provide more information on the overall burden of ANFR CO poisoning including prognosis of those admitted to hospital.

## Conclusions

This work has shown that the numbers of people admitted to hospital with ANFR CO poisoning in England are larger than previously estimated and does not appear to be reducing. The burden of ANFR CO poisoning is preventable, and these results highlight differences that could be used to focus interventions on to groups who may most benefit from targeted public health interventions, for example older individuals, those with young children, deprived areas and those living in areas with colder winters. A public health surveillance programme to identify common sources of CO that give rise to potential harmful human exposure as well as providing geographical and other relevant information could be developed in England and other countries, similar to those already in operation in France and a few USA states. This approach has long been accepted practice in the surveillance of infectious diseases and would raise awareness as well as provide a relevant evidence base to help plan preventative strategies. The implementation of effective measures to reduce the morbidity and mortality caused by exposure to CO would also reduce an economic burden on health services.

## Supplementary data

Supplementary data are available at *PUBMED* online.

## Funding

The research was funded by the National Institute for Health Research Health Protection Research Unit (NIHR HPRU) in Health Impact of Environmental Hazards at King's College London in partnership with Public Health England (PHE). The views expressed are those of the author(s) and not necessarily those of the NHS, the NIHR, the Department of Health or Public Health England.

## Supplementary Material

Supplementary Data
