# Supplementary material for: Analysis of hospital admissions due to accidental non-fire-related carbon monoxide poisoning in England, between 2001 and 2010
Source: J Public Health (Oxf). 2015 Mar 9;38(1):76–83. doi: 10.1093/pubmed/fdv026 (PMC4750524; doi:10.1093/pubmed/fdv026)
Supplement: Supplementary Data [file supp_38_1_76__index.html]

Analysis of hospital admissions due to accidental non-fire-related carbon monoxide poisoning in England, between 2001 and 2010 — Analysis of hospital admissions due to accidental non-fire-related carbon monoxide poisoning in England, between 2001 and 2010 — Analysis of hospital admissions due to accidental non-fire-related carbon monoxide poisoning in England, between 2001 and 2010 — Supplementary Data 

# Analysis of hospital admissions due to accidental non-fire-related carbon monoxide poisoning in England, between 2001 and 2010

## Supplementary Data

Supplementary Data

**Files in this Data Supplement:**

- Supplementary Data - Docx file
