# Supplementary material for: Analysis of hospital admissions due to accidental non-fire-related carbon monoxide poisoning in England, between 2001 and 2010
Source: J Public Health (Oxf). 2015 Mar 9;38(1):76–83. doi: 10.1093/pubmed/fdv026 (PMC4750524; doi:10.1093/pubmed/fdv026)
Supplement: Supplementary Data [file supp_fdv026_fdv026supp.docx]

# Appendix:

**Supplemental table 1: Carbon monoxide admissions (2001-2010, England) by sex and intent, primary diagnosis of T58 only**

|  | **Accidental-X47 n (%)** | **Intentional-X67 n (%)** | **Unknown ^1^ n (%)** | **Total n (%)** |
| --- | --- | --- | --- | --- |
|  | **Including fire related codes** | | | |
| **Female** | 1078 (46.5) | 281 (16.2) | 198 (43.9) | 1557 (34.5) |
| **Male** | 1241 (53.5)^2^ | 1457 (83.8)^2^ | 253 (56.1)^2^ | 2951 (65.5) |
| **Total** | 2319 (100) | 1738 (100) | 451 (100)^3^ | 4508 (100) |
|  | **Excluding fire related codes** | | | |
| **Female** | 1070 (46.4) | 281 (16.2) | 136 (52.6) | 1487 (34.2) |
| **Male** | 1234 (53.6)^2^ | 1455 (83.8)^2^ | 170 (47.4)^2^ | 2859 (65.8) |
| **Total1** | 2304 (100) | 1736 (100) | 306 (100)^4^ | 4346 (100) |

^1^ Combination of “T58+Y17 – Undetermined intent” and T58 with no intent code provided

^2^ Difference between sexes statistically significant : Χ**^2^** p<0.001

^3^ Of these 394 were admissions with T58 but no other intent code

^4^ Of these 249 were admissions with T58 but no other intent code

**Supplemental Figure 1: Smoothed relative risk of hospital admissions for accidental non-fire related (ANFR) CO hospital admission at local authority and district level with and without adjustment for deprivation (Carstairs index).**

**
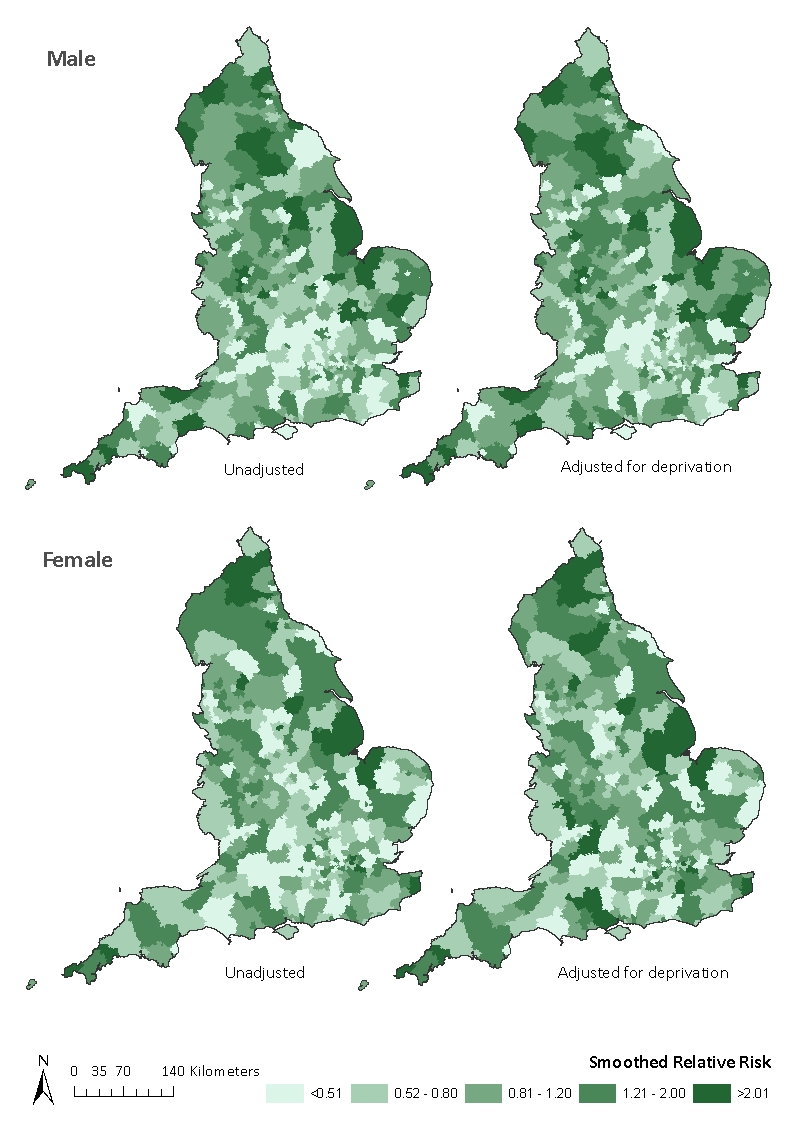
**
